# The SCOP database in 2020: expanded classification of representative family and superfamily domains of known protein structures

**DOI:** 10.1093/nar/gkz1064

**Published:** 2019-11-14

**Authors:** Antonina Andreeva, Eugene Kulesha, Julian Gough, Alexey G Murzin

**Affiliations:** 1 MRC Laboratory of Molecular Biology, Francis Crick Avenue, Cambridge CB2 0QH, UK; 2 EKMM Limited, Cambridge CB1 9AZ, UK

## Abstract

The Structural Classification of Proteins (SCOP) database is a classification of protein domains organised according to their evolutionary and structural relationships. We report a major effort to increase the coverage of structural data, aiming to provide classification of almost all domain superfamilies with representatives in the PDB. We have also improved the database schema, provided a new API and modernised the web interface. This is by far the most significant update in coverage since SCOP 1.75 and builds on the advances in schema from the SCOP 2 prototype. The database is accessible from http://scop.mrc-lmb.cam.ac.uk.

## INTRODUCTION

The SCOP database is a classification that organises proteins of known three-dimensional structure according to their structural and evolutionary relationships (1). It was established in 1994 at MRC LMB and CPE in Cambridge and over the years has attracted a broad range of users, thus becoming a valuable resource in different areas of protein research.

The classification of proteins in SCOP has been constructed mainly manually by visual inspection and analysis. Like the Linnaean taxonomy, SCOP was created as a hierarchy in which discrete units, domains, were organized into different levels on the basis of their common structural features and evolutionary relationships. Depending on the degree of evolutionary divergence, protein domains were organized into families and superfamilies. These were further grouped into structural folds, which were not necessarily indicative of a common evolutionary origin and classes reflecting their secondary structural content.

The primary purpose of SCOP was to assist experimental structural biologists in the analysis and exploration of protein structures similar to their proteins of research. The wide range of protein structural and evolutionary relationships were also exploited by computational biologists for benchmarking and evaluation of protein structure comparison and prediction methods. The simple hierarchical classification facilitated the development of many tools and algorithms and it was successfully used by many applications. The database was applied to other areas of protein research such as protein structure prediction and large-scale genome analyses and annotations. SCOP has also been used for prediction of protein–protein interactions, mapping protein structure with enzymatic activity and other studies aiming to understand the complexity of protein repertoire.

SCOP has always been a research project and its value arose from the body of quality data incorporating a vast knowledge and encyclopedic expertise on proteins and their relations. Each grouping in the classification was a product of a careful, systematic analysis of protein structures and a detailed knowledge of protein function and evolution. Many distant evolutionary relationships between proteins were first discovered during their analysis for classification in SCOP. Some of these have never been described in the literature and thus the SCOP database has become a repository for many interesting research findings.

The work on SCOP 1.75 concluded in 2014 and we then initiated the development of the SCOP 2 prototype (2) in an attempt to capture new discoveries and findings or to recreate complex scenarios of protein evolution. The prototype was designed to retain the best features of SCOP and provide groupings of proteins based not only on their structural features but also on their common evolutionary origin. Depending on the degree of evolutionary divergence and structural similarity, proteins were organized into distinct levels that instead of a strict tree-like hierarchy, were allowed to form more complex multiple parental relationships. The working prototype contained only a small fraction of the available structural data pending users’ feedback essential for its future development and expansion.

Here we describe the new developments and update of the SCOP database. We have developed a new version of SCOP by expanding and greatly simplifying the SCOP 2 prototype design. We have undertaken a number of efforts to improve the database schema, develop a new dynamic web-interface but most importantly, increase the SCOP coverage of PDB structures.

## SCOP DEVELOPMENT AND UPDATE

We have redesigned the SCOP 2 prototype schema and expanded the database by incorporating the SCOP version 1.75 data. We have further undertaken a significant update of protein structures by ensuring that the growth of the database reflects increased diversity and quality rather than solely the increase in data quantity. Our main focus was on novelty and discovery of new and more distant evolutionary relationships, particularly these that are not detectable with advanced automated methods. In addition, we have steadily continued adding new PDB entries to already existing SCOP families while concurrently revising and improving their classification. Compared to SCOP 1.75, the number of families and superfamilies has grown substantially. At the time of writing the current SCOP database contains 5058 families and 2455 superfamilies compared to 3902 families and 1962 superfamilies in SCOP 1.75 (see Table 1 for more details). The classification levels currently organise more than 40 000 non-redundant domains that represent nearly 500 000 protein structures.

**Table 1.** Progress of the SCOP classification compared to SCOP version 1.75

Number	**SCOP 2**	**SCOP 1.75**
***Folds***	1388	1195
***IUPRs***	17	n.a.
***Hyperfamilies***	15	n.a.
***Superfamilies***	2455	1962
***Families***	5060	3902
***Interrelationships***	46	n.a.

The current SCOP classification schema has taken advantage of both SCOP 1.75 and SCOP 2 prototype, and in essence, it is a merger of these two developments. More details about the design, content and the rationale behind the current classification are provided below.

### Current SCOP classification structure

Two evolutionary levels: *family* and *superfamily* are at the heart of the current SCOP classification. *Family* groups closely related proteins with a clear evidence for their evolutionary origin while *superfamily* brings together more distantly related protein domains. As these relationships can sometimes span structural regions of different size, we provide domain boundaries for both, *family* and *superfamily* levels.

*Superfamilies* are grouped into distinct *folds* on the basis of the global structural features shared by the majority of their members. These features are the composition of the secondary structures in the domain core, their architecture and topology. Fold is an attribute of a *superfamily* but the constituent *families* of some *superfamilies* that have evolved distinct structural features can belong to a different fold. *Superfamilies* of proteins or protein regions that do not adopt globular folded structure are grouped in *IUPR*s (Intrinsically Unstructured Protein Region). Some of these proteins exist in an ensemble of different conformations or are unstructured in free state but adopt an ordered conformation upon binding to other macromolecules.

*Folds* and *IUPRs* with different secondary structural content are placed into one of the five different structural classes. These include all-alpha and all-beta proteins, containing predominantly alpha-helices and beta-strands, respectively, and ‘mixed’ alpha and beta classes (a/b) and (a+b) with respectively alternating and segregated alpha-helices and beta-strands, and the fifth class of small proteins with little or no secondary structures. *Folds* and *IUPRs* are also grouped based on their *protein type*, into four groups: soluble, membrane, fibrous and intrinsically disordered. Each of these types to a large extent correlates with characteristic sequence and structural features.

All classification levels in the current SCOP database are obligatory. Thus, a protein domain belongs to a family and superfamily that are organized into folds or IUPRs grouped into a particular class and of particular type.

### Building up the SCOP version 2 content

The SCOP version 2 builds on the advances in schema from the SCOP 2 prototype. To improve the database usability and facilitate the future updates, the SCOP 2 prototype design was simplified by reducing the levels of classification to: *type*, *class*, *fold/IUPR*, *superfamily* and *family*. Most of the SCOP 1.75 classification data were directly integrated on the SCOP 2 platform. Some classes in SCOP 1.75 such as multi-domain proteins, coiled coil, membrane proteins, low resolution protein structures, peptides and designed proteins that were not true structural classes were reclassified or excluded. Most of the SCOP 1.75 domains belonging to multi-domain proteins class were reclassified in SCOP 2. If necessary, entries were split into their constituent domains that were then placed in a new or already existing SCOP fold. All folds and their constituent members belonging to coiled coil and membrane proteins classes were reassigned to their correct structural class and type thus resolving their inconsistent classification in SCOP 1.75. Entries classified in peptides and designed proteins classes were not included in SCOP 2. Prior to the integration of the SCOP 1.75 domain assignments, representative structures were selected on the basis of their coverage of UniProtKB entry, resolution, number of visible residues etc. Boundaries of all SCOP 1.75 domains were assigned to representative PDB and UniProtKB entries that resulted in a refinement of many domain definitions. Following the merger, the SCOP database was updated with representative protein structures prioritising those belonging to Pfam families containing structurally characterized proteins that were not classified in SCOP 1.75.

### Protein relationships in SCOP

**Figure 1.**
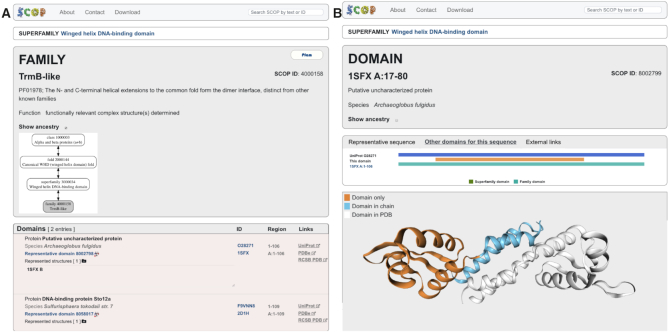
Trivial protein relationships in the current SCOP classification. These are exemplified by TrmB-like family of transcriptional regulators (SCOP ID 4000158) that are related to protein domains members of the SCOP ‘Winged helix DNA-binding domains’ superfamily (SCOP ID 3000034). Their classification is very similar to the SCOP 1.75 classification. (**A**) SCOP family page showing details of the node classification and annotation. A clickable ancestry chart displays the hierarchical relations between different nodes and allows navigating and exploring their classification. At the bottom of the page all relevant information about the constituent family domains is listed. (**B**) SCOP superfamily domain page of a member of ‘Winged helix DNA-binding domains’ superfamily (SCOP ID 3000034) showing details of its sequence and structure. On the sequence viewer both, the family and superfamily domains are displayed and demonstrate their differences. The superfamily domain is smaller than the family domain as it defines the evolutionary conserved core of this superfamily.

**Figure 2.**
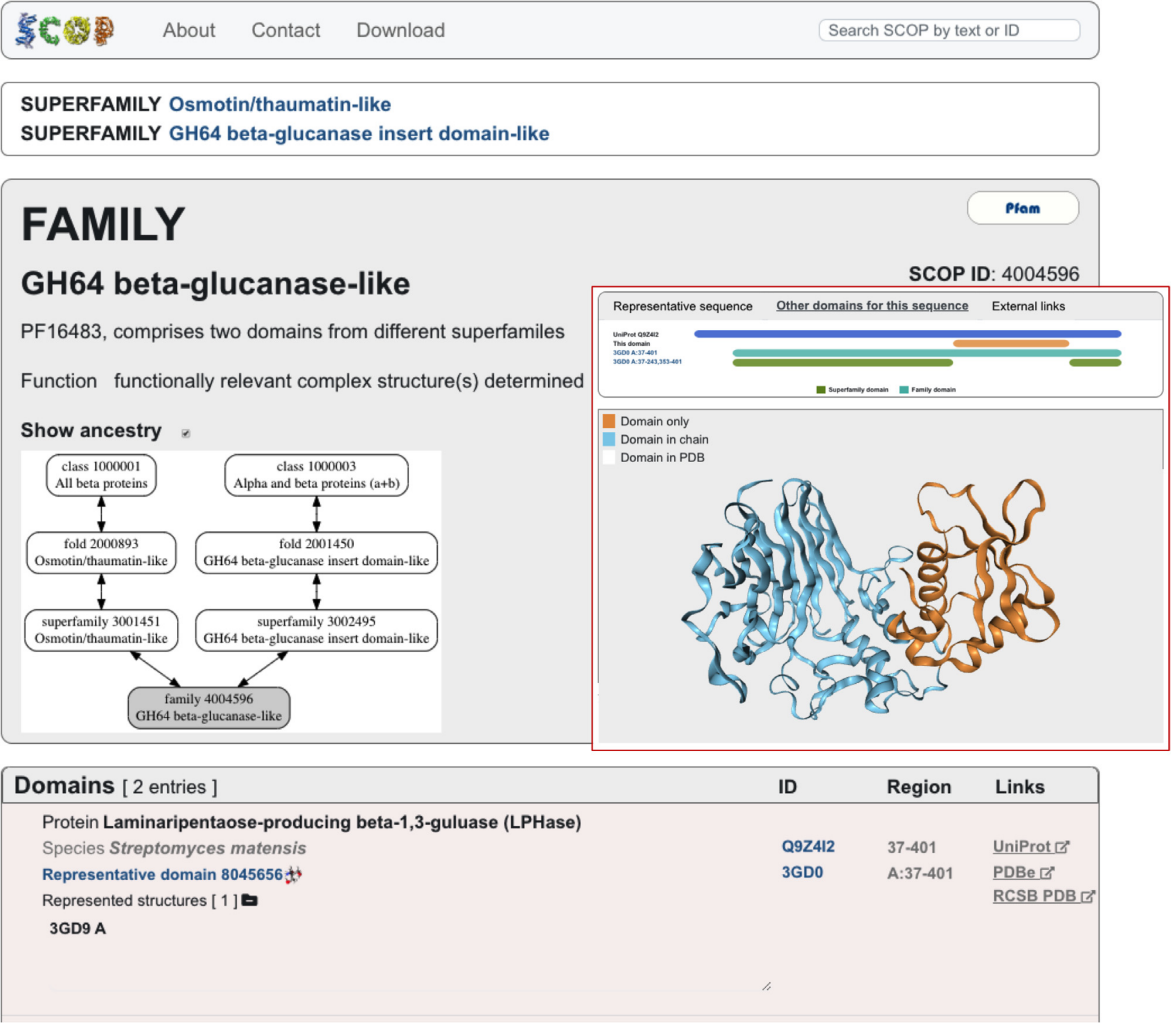
SCOP family that comprises two domains each of which a member of a distinct superfamily. The glycoside hydrolase family 64 (SCOP ID 4004596) domain spans over two structural domains, one of which belongs to the ‘Osmotin/thaumatin-like’ superfamily (SCOP ID 3001451) and the other, of a novel fold, classified into its own superfamily (SCOP ID 3002495) (23).

**Figure 3.**
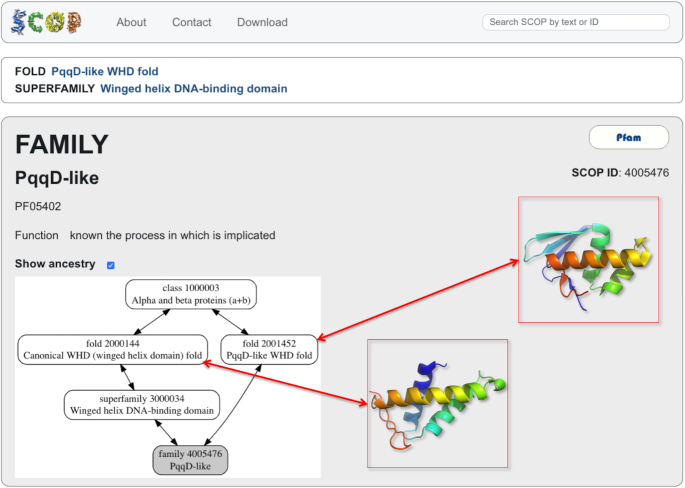
SCOP family with a fold distinct from the fold of the other superfamily domains. The ‘PqqD-like’ family of PQQ biosynthesis enzymes belongs to the superfamily of ‘Winged helix DNA-binding domains’ (SCOP ID 3000034) but it has evolved a globally different fold from the other superfamily members.

Most of the protein relationships in the current SCOP classification are trivial and can be described as a hierarchical tree in which protein family and superfamily domains are grouped according to their structural similarity and evolutionary divergence and their boundaries correlate with each other (Figure 1). Some protein relationships in the classification can be more complex but these are limited to two possible scenarios. The first scenario is when a family domain spans two or more structural domains each of which belong to a distinct superfamily, e.g. the combination and arrangement of these domains evolved within, and is typical for this family of proteins (Figure 2). The second scenario is when a family contains domains that are topologically more similar to another distinct fold than to the fold of the other superfamily domains, e.g. the superfamily brings together evolutionary related proteins with distinct folds. The evolutionary and structural relationships of this family are non-hierarchical (Figure 3, Supplementary Figure S1). In summary, to a large extent the current SCOP classification is a hierarchical tree and the more complex, multiple parental relationships follow the rules described above.

### SCOP domains – definitions and boundaries

The database is now built as a classification of non-redundant protein domains. A representative is selected based on its sequence (UniProtKB) and structure (PDB) and used for SCOP classification. Thus, the SCOP domain boundaries are assigned to both, the PDB and UniProtKB entry. The manual classification of this selected representative is then automatically extended to related entries using SIFTS (3). SIFTS is a resource that provides residue-level cross-references between protein sequences in UniProtKB (4) and 3D atomic models of these proteins in PDB (5). Using UniProtKB sequence as a means for clustering provides an objective criterion for a selection of a non-redundant set of protein domains.

Domain definitions are provided for the two main levels of the SCOP classification, family and superfamily, and the domain boundaries for each of these can coincide or differ. There are two main reasons for providing different boundaries at *family* and *superfamily* level. The first is when the combination and arrangement of two or more domains is conserved within the family. The interface between these domains usually engage highly conserved residues that define a family specific sequence fingerprint. Each of the constituent domains can be distantly related and structurally similar either to other domains from functionally distinct proteins or to each other. The family domain boundaries define the conserved multidomain region whereas the superfamily domains span over the individual domains (Figure 2). The other reason for different domain boundaries is when the protein domain is highly elaborated with additional secondary structures forming a substructure. This substructure, however, hasn’t been observed in other proteins and does not define an evolutionary conserved domain. The family domain defines the entire region including the substructure whereas the superfamily domain defines the smaller evolutionary conserved core domain (Figure 1B). The rationale behind these different definitions is that sequence fragments of different sizes retrieve different matches and thus sequence libraries produced using family and superfamily domain sequences can be complementary and may increase the sensitivity of sequence searches. They may also provide an alternative solution to some challenging problems particularly associated with detection and prediction of protein domains consisting of two or more discontinuous segments.

### Functional and structural annotations

Several groupings, introduced in the SCOP 2 prototype, are becoming part of the annotations in the current SCOP classification. These include hyperfamily and various relationships such as internal structural repeats, common motifs and subfolds. In addition to the classification data, we provide manual functional and structural annotations. Functional annotations are defined for families and describe the extent to which a SCOP family is functionally characterised. Additional annotation of the SCOP nodes and domains is provided using a controlled vocabulary. This allows easy retrieval of a subset of proteins with a given structural feature or automatic processing of the classification data.

### New findings during the classification update

During the analysis of protein structures for their classification in SCOP, there were several interesting findings of previously unseen topologies and architectures, novel structural features and unexpected evolutionary relationships. Amongst these were the novel type of protein architecture observed in the β-braid fold (6) (Supplementary Figure S2) and the distant evolutionary relationship between the members of the new β-prism III and Tricorn folds, both exhibiting pseudo three fold symmetry (7,8) (Supplementary Figure S3).

## SCOP AND OTHER PROTEIN DATABASES

### SCOP and UniProtKB

The current SCOP classification provides domain boundaries for a representative PDB structure and for a UniProtKB sequence. It relies on the SIFTS resource to establish sequence cross-reference between PDB and UniProtKB for propagation of the representative data to other entries in PDB. Providing SCOP domain boundaries on UniProtKB sequence has several advantages. It allows the exploitation and transfer of the biological annotations available from UniProtKB. It allows also to present the SCOP classification in a context of the entire protein. This may be beneficial for molecular modelling of entire proteins in large complexes as it allows easy to assemble structurally characterized parts of a protein of interest.

Undoubtedly, the biggest advantage is the generation of the SCOP sequence libraries. The sequence libraries can be easily produced using the UniProtKB sequence and thus providing a non-redundant set of protein domain sequences. This SCOP sequence library is built using only the natural protein sequences, excluding the sequences with engineered mutations, artificial inserts and sequence tags. Previously, the SCOP domain sequences were derived from the PDB files and together with the representative subsets were provided by Astral (9). We recommend our users to take advantage of the SCOP version 2 sequence libraries as they aim to address problems associated with non-natural protein sequences that can affect the outcome of sequence search results. The residue-level cross-reference between the SCOP domain sequence derived from UniProtKB entry and any PDB structure can be easily established using SIFTS.

### SCOP and PDB redundancy – representative domains and represented structures

As the single worldwide repository for macromolecular structures, the PDB contains a body of data with a considerable redundancy in regard to both sequence and structure. For example clustering of PDB protein chains with more than 20 residues using blastclust and based on 90% coverage and 100% identity results in more than five-fold reduction of the set. Clustering at 90% identity reduces the data nearly ten-fold. Therefore many of the protein structure resources provide non-redundant sets of their data clustered usually by 30%, 50% and 90% identity. The disadvantage of a clustering of this type is that there is no objective selection of the cluster representative and that sequences of engineered mutant proteins or fragments of a protein can appear in different clusters than the native protein.

We deal with the PDB redundancy by selecting a representative PDB structure for a particular UniProtKB entry. The cluster of represented structures for the representative SCOP domain is then derived using the SIFTS mapping. Thus we believe we provide unbiased clustered set of the SCOP data. Those who programmatically use the SCOP data can retrieve the up-to-date conformational ensemble of PDB structures for a given SCOP domain using the latest SIFTS mapping, regardless of the database update.

### SCOP and Pfam database

There has been a long lasting partnership between the SCOP and Pfam (10) databases. Many Pfam families originate from corresponding SCOP families, while the classification of new protein structures in SCOP takes into account their Pfam assignment. The relationship between the SCOP and Pfam families is not explicit. There are differences in boundaries and members of the families. One Pfam family can correspond to two or more SCOP families and vice versa. For example, the CDGSH iron sulphur domains (CISD), members of a single Pfam family (PF09360), are found to adopt several distinct folds (11). Each different structural type CISD is classified into a different SCOP family (Supplementary Figure S1). The SCOP family of RecA/Rad51/KaiC-like ATPases (SCOP ID 4004007) brings together members of several related Pfam families with very similar structures. These complex relationships between SCOP and Pfam families are due to genuine design differences and technical underpinnings of these databases. We continue annotating the mapping between the SCOP and Pfam families. These curated relationships are shown in the comment field at the family level and as a hyperlink to the Pfam database.

### SCOP and SUPERFAMILY database

The SUPERFAMILY database uses a library of hidden Markov models to annotate protein sequences with structural domains (12). The HMM 1.75 SUPERFAMILY library was build using sequences of domains classified at the superfamily level of the SCOP legacy 1.75 database. Recently, the HMM 2.0 library was built to provide improved structural and functional annotations for the millions of protein sequences obtained from major resources including UniProtKB and NCBI complete genome collection (13). In addition to HMM 1.75 models, the HMM 2.0 library contained models built from the domain sequences taken from other structural classification databases such as SCOPe (14), CATH (15) and ECOD (16) as well as from the entire protein chain sequences taken from the PDB. In the current SCOP web interface, we provide a link in every superfamily page to effectively integrate the current SCOP superfamily classification with its predicted superfamily domain annotations found in all protein sequences of all genomes in the SUPERFAMILY database. The example link http://supfam.org/allcombs/3001658 demonstrates the connection of the SCOP Ferritin-like superfamily to its corresponding genome annotations in the SUPERFAMILY database.

The work on adding HMM models representing newly classified SCOP superfamily sequences to the SUPERFAMILY HMM 2.0 library is in progress. The updated HMM library will also incorporate the changes of the SCOP classification in the SUPERFAMILY database. This addition is expected to considerably improve the structural coverage of the genomic data.

## NEW WEB INTERFACE

The SCOP database is available over the web from http://scop.mrc-lmb.cam.ac.uk. A new dynamic web interface was developed allowing easy-to-navigate way of accessing the SCOP data. It has been designed to facilitate both detailed searching and browsing of the database. The SCOP search engine supports queries with free text, PDB, UniProtKB and SCOP node identifier. When browsing SCOP, there are two ways of entry into the classification: by structural class or by protein type. Users can explore the classification data by navigating through different levels of folds, superfamilies and families. The superfamily and family pages list the corresponding node annotation and their constituent domains. All relevant information about a particular SCOP protein domain is displayed on a page showing details of its sequence and structure (Figure 1B). A sequence viewer allows the user to allocate the SCOP domain on the entire UniProtKB protein sequence as well as to retrieve all domains for this protein, classified in SCOP. The structure of the domain is visualized using NGL viewer (17) and displayed within the context of a given PDB entry. A clickable ancestry chart allows to explore the classification of the domain and navigate through different classification levels. The domain page also provides a set of links to external resources such as CATH (15), ECOD (16), Protein contacts atlas (18), PDBe-KB and PDBe-PISA (19) as well as a range of resources for structure comparison and searches such as DALI (20), FATCAT (21), TOPMATCH (22) and PDBeFold (19) allowing users to compare and search for structural similarities using different structure comparison algorithms.

To all nodes are assigned new seven-digit unique identifiers that provide a stable reference to the database. Nearly all SCOP data are accessible via Representation State Transfer (REST) protocol. The SCOP REST API web service enables fast and easy programming language-agnostic access to the SCOP classification and SCOP domains. The data can be requested with a simple HTTP request and returned in JSON. The service is accessible at http://scop.mrc-lmb.cam.ac.uk/api/.

## CONCLUSION

Since it was created, the development of SCOP has been always guided by its users feedback and needs. The automation in crystallography and advances in cryo-electron microscopy open a new era in structural biology and with it come new demands for data suitable for modelling of large proteins and protein complexes. We have considerably expanded and updated the SCOP database and implemented new features in attempt to meet some of these demands. To better serve the expanded and growing data we have developed a new user interface and API which allow to make the SCOP data readily accessible in a flexible manner. In addition to a range of new annotations, we introduced new functionalities that support relatively easy retrieval and assembly of independently determined, structurally characterized parts of proteins of interest. We shall continue updating the database and providing regular releases while working on steadily increasing the coverage of structural data and adding new functionalities to the web interface.

## Supplementary Material

gkz1064_Supplemental_File

## References

[1] Murzin A.G., Brenner S.E., Hubbard T., Chothia C. SCOP: a structural classification of proteins database for the investigation of sequences and structures. J. Mol. Biol. 1995; 247:536–540. 7723011 10.1006/jmbi.1995.0159

[2] Andreeva A., Howorth D., Chothia C., Kulesha E., Murzin A.G. SCOP2 prototype: a new approach to protein structure mining. Nucleic Acids Res. 2014; 42:D310–D314. 24293656 10.1093/nar/gkt1242 PMC3964979

[3] Dana J.M., Gutmanas A., Tyagi N., Qi G., O’Donovan C., Martin M., Velankar S. SIFTS: updated Structure Integration with Function, Taxonomy and Sequences resource allows 40-fold increase in coverage of structure-based annotations for proteins. Nucleic Acids Res. 2019; 47:D482–D489. 30445541 10.1093/nar/gky1114 PMC6324003

[4] UniProt Consortium UniProt: a worldwide hub of protein knowledge. Nucleic Acids Res. 2019; 47:D506–D515. 30395287 10.1093/nar/gky1049 PMC6323992

[5] wwPDB consortium Protein Data Bank: the single global archive for 3D macromolecular structure data. Nucleic Acids Res. 2019; 47:D520–D528. 30357364 10.1093/nar/gky949 PMC6324056

[6] Conrady D.G., Wilson J.J., Herr A.B. Structural basis for Zn2+-dependent intercellular adhesion in staphylococcal biofilms. Proc. Natl. Acad. Sci. U.S.A. 2013; 110:E202–E211. 23277549 10.1073/pnas.1208134110 PMC3549106

[7] Rosado C.J., Buckle A.M., Law R.H., Butcher R.E., Kan W.T., Bird C.H., Ung K., Browne K.A., Baran K. et al. A common fold mediates vertebrate defense and bacterial attack. Science. 2007; 317:1548–1551. 17717151 10.1126/science.1144706

[8] Lukoyanova N., Kondos S.C., Farabella I., Law R.H., Reboul C.F., Caradoc-Davies T.T., Spicer B.A., Kleifeld O., Traore D.A., Ekkel S.M. et al. Conformational changes during pore formation by the perforin-related protein pleurotolysin. PLoS Biol. 2015; 13:e1002049. 25654333 10.1371/journal.pbio.1002049 PMC4318580

[9] Chandonia J.M., Hon G., Walker N.S., Lo Conte L., Koehl P., Levitt M., Brenner S.E. The ASTRAL Compendium in 2004. Nucleic Acids Res. 2004; 32:D189–D192. 14681391 10.1093/nar/gkh034 PMC308768

[10] El-Gebali S., Mistry J., Bateman A., Eddy S.R., Luciani A., Potter S.C., Qureshi M., Richardson L.J., Salazar G.A., Smart A. et al. The Pfam protein families database in 2019. Nucleic Acids Res. 2019; 47:D427–D432. 30357350 10.1093/nar/gky995 PMC6324024

[11] Lin J., Zhang L., Lai S., Ye K. Structure and molecular evolution of CDGSH iron-sulfur domains. PLoS One. 2011; 6:e24790. 21949752 10.1371/journal.pone.0024790 PMC3174974

[12] Gough J., Karplus K., Hughey R., Chothia C. Assignment of homology to genome sequences using a library of hidden Markov models that represent all proteins of known structure. J. Mol. Biol. 2001; 313:903–919. 11697912 10.1006/jmbi.2001.5080

[13] Pandurangan A.P., Stahlhacke J., Oates M.E., Smithers B., Gough J. The SUPERFAMILY 2.0 database: a significant proteome update and a new webserver. Nucleic Acids Res. 2019; 47:D490–D494. 30445555 10.1093/nar/gky1130 PMC6324026

[14] Fox N.K., Brenner S.E., Chandonia J.M. SCOPe: Structural Classification of Proteins–extended, integrating SCOP and ASTRAL data and classification of new structures. Nucleic Acids Res. 2014; 42:D304–D309. 24304899 10.1093/nar/gkt1240 PMC3965108

[15] Dawson N.L., Lewis T.E., Das S., Lees J.G., Lee D., Ashford P., Orengo C.A., Sillitoe I. CATH: an expanded resource to predict protein function through structure and sequence. Nucleic Acids Res. 2017; 45:D289–D295. 27899584 10.1093/nar/gkw1098 PMC5210570

[16] Cheng H., Schaeffer R.D., Liao Y., Kinch L.N., Pei J., Shi S., Kim B.H., Grishin N.V. ECOD: an evolutionary classification of protein domains. PLoS Comput. Biol. 2014; 10:e1003926. 25474468 10.1371/journal.pcbi.1003926 PMC4256011

[17] Rose A.S., Bradley A.R., Valasatava Y., Duarte J.M., Prlic A., Rose P.W. NGL viewer: web-based molecular graphics for large complexes. Bioinformatics. 2018; 34:3755–3758. 29850778 10.1093/bioinformatics/bty419 PMC6198858

[18] Kayikci M., Venkatakrishnan A.J., Scott-Brown J., Ravarani C.N.J., Flock T., Babu M.M. Visualization and analysis of non-covalent contacts using the Protein Contacts Atlas. Nat. Struct. Mol. Biol. 2018; 25:185–194. 29335563 10.1038/s41594-017-0019-z PMC5837000

[19] Cook C.E., Lopez R., Stroe O., Cochrane G., Brooksbank C., Birney E., Apweiler R. The European Bioinformatics Institute in 2018: tools, infrastructure and training. Nucleic Acids Res. 2019; 47:D15–D22. 30445657 10.1093/nar/gky1124 PMC6323906

[20] Holm L., Laakso L.M. Dali server update. Nucleic Acids Res. 2016; 44:W351–W355. 27131377 10.1093/nar/gkw357 PMC4987910

[21] Ye Y., Godzik A. Flexible structure alignment by chaining aligned fragment pairs allowing twists. Bioinformatics. 2003; 19:ii246–ii255. 14534198 10.1093/bioinformatics/btg1086

[22] Sippl M.J., Wiederstein M. Detection of spatial correlations in protein structures and molecular complexes. Structure. 2012; 20:718–728. 22483118 10.1016/j.str.2012.01.024 PMC3320710

[23] Wu H.M., Liu S.W., Hsu M.T., Hung C.L., Lai C.C., Cheng W.C., Wang H.J., Li Y.K., Wang W.C. Structure, mechanistic action, and essential residues of a GH-64 enzyme, laminaripentaose-producing beta-1,3-glucanase. J. Biol. Chem. 2009; 284:26708–26715. 19640850 10.1074/jbc.M109.010983 PMC2785358

