# Supplementary material for: The SCOP database in 2020: expanded classification of representative family and superfamily domains of known protein structures
Source: Nucleic Acids Res. 2019 Nov 14;48(D1):D376–82. doi: 10.1093/nar/gkz1064 (PMC7139981; doi:10.1093/nar/gkz1064)
Supplement: gkz1064_Supplemental_File [file gkz1064_supplemental_file.pdf]

## SUPPLEMENTARY MATERIAL

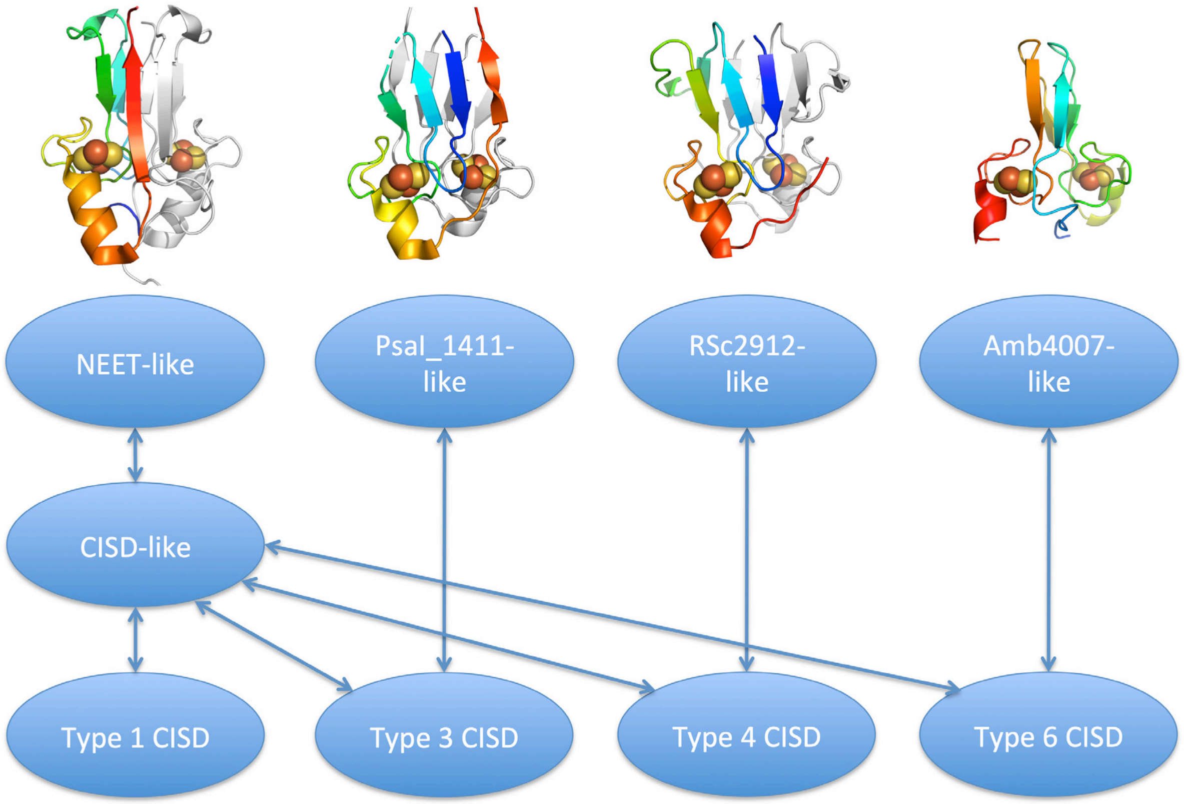


**Supplementary Figure 1. Structural and evolutionary relationships of homologous families with different folds.**

The CISD families of different types, sharing CDGSH iron sulphur cluster-binding motif, are classified into the same CISD-like superfamily. The fold of the largest type 1 family is selected as representative for this superfamily, whereas each other family is assigned to a different fold. The classification of these families exemplifies a complex scenario of non-hierarchical evolutionary and structural relationships.

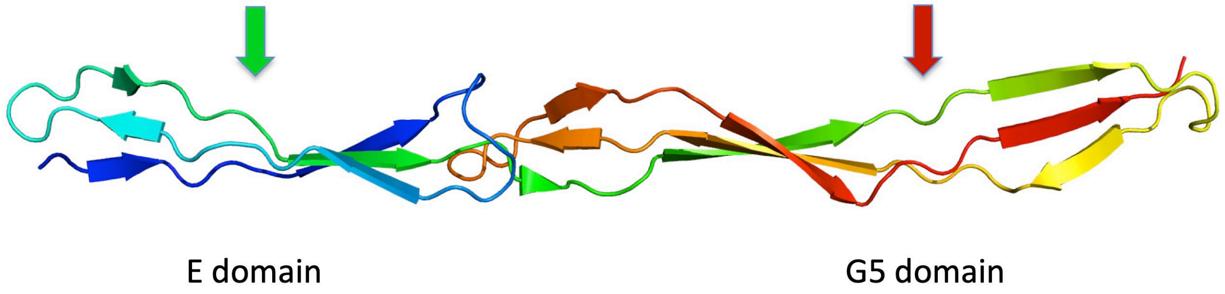


**Supplementary Figure 2**. **Novel type of protein architecture in the β-braid fold (SCOP ID 2001419).**

This small fold, lacking a hydrophobic core, is made of three interlaced extended segments, capped on both ends by three-stranded β-sheets with different strand orders. This fold is found in the related G5 domain and E domain families, which are present together in Staphylococcal SasG and Aap proteins (*1*), a section of which is shown here as a rainbow-coloured cartoon. Interlacing sections of both domains are indicated by bold arrows coloured green and red, as their C-terminal segments. Before the determination of its structure, the smaller E domain was thought to be unstructured.

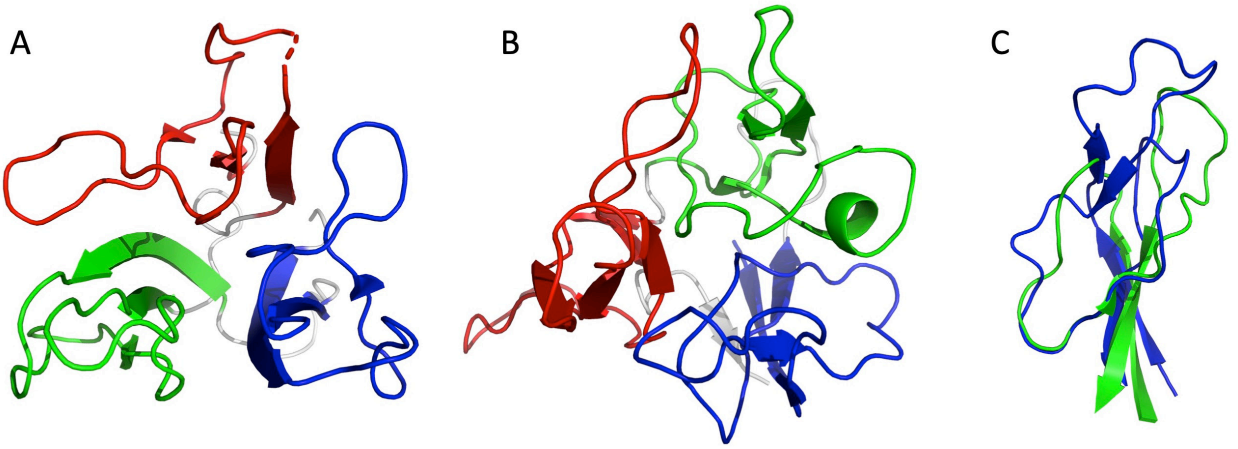


**Supplementary Figure 3. Intra- and interrelationships of two different new folds**

The β-prism III (A; SCOP ID 2001273) and Tricorn (B; SCOP ID 2001279) folds are new folds exhibiting pseudo three fold symmetry. Both folds are composed of three compact motifs shown in different colours: N-terminal in blue, middle in green and C-terminal in red. Individual motifs from the different folds have similar topologies (C; showing the green motif of A superimposed on the blue motif of B), but are assembled differently in different folds. The β-prism III and Tricorn domains are found in the MACPF proteins in the same position C-terminal to the pore-forming domains, suggesting their very remote evolutionary relationship (*2, 3*)


1. Conrady, D. G., Wilson, J. J., and Herr, A. B. (2013) Structural basis for Zn2+-dependent intercellular adhesion in staphylococcal biofilms, *Proc Natl Acad Sci U S A 110*, E202-211.
2. Rosado, C. J., Buckle, A. M., Law, R. H., Butcher, R. E., Kan, W. T., Bird, C. H., Ung, K., Browne, K. A., Baran, K., Bashtannyk-Puhalovich, T. A., Faux, N. G., Wong, W., Porter, C. J., Pike, R. N., Ellisdon, A. M., Pearce, M. C., Bottomley, S. P., Emsley, J., Smith, A. I., Rossjohn, J., Hartland, E. L., Voskoboinik, I., Trapani, J. A., Bird, P. I., Dunstone, M. A., and Whisstock, J. C. (2007) A common fold mediates vertebrate defense and bacterial attack, *Science 317*, 1548-1551.
3. Lukoyanova, N., Kondos, S. C., Farabella, I., Law, R. H., Reboul, C. F., Caradoc-Davies, T. T., Spicer, B. A., Kleifeld, O., Traore, D. A., Ekkel, S. M., Voskoboinik, I., Trapani, J. A., Hatfaludi, T., Oliver, K., Hotze, E. M., Tweten, R. K., Whisstock, J. C., Topf, M., Saibil, H. R., and Dunstone, M. A. (2015) Conformational changes during pore formation by the perforin-related protein pleurotolysin, *PLoS Biol 13*, e1002049.
